# Randomized controlled trial to evaluate the effects of progressive resistance training compared to progressive muscle relaxation in breast cancer patients undergoing adjuvant radiotherapy: the BEST study

**DOI:** 10.1186/1471-2407-13-162

**Published:** 2013-03-28

**Authors:** Karin Potthoff, Martina E Schmidt, Joachim Wiskemann, Holger Hof, Oliver Klassen, Nina Habermann, Philipp Beckhove, Juergen Debus, Cornelia M Ulrich, Karen Steindorf

**Affiliations:** 1 Department of Radiation Oncology, University of Heidelberg Medical Center, Im Neuenheimer Feld 400, Heidelberg 69120, Germany; 2 Unit of Physical Activity and Cancer, German Cancer Research Center, Im Neuenheimer Feld 280, Heidelberg 69120, Germany; 3 Department of Preventive Oncology, National Center for Tumor Diseases, Im Neuenheimer Feld 460, Heidelberg 69120, Germany; 4 Department of Medical Oncology, National Center for Tumor Diseases, Im Neuenheimer Feld 460, Heidelberg 69120, Germany; 5 Division of Translational Immunology, National Center for Tumor Diseases, Im Neuenheimer Feld 460, Heidelberg 69120, Germany

## Abstract

**Background:**

Cancer-related fatigue (CRF) is one of the most common and distressing side effects of cancer and its treatment. During and after radiotherapy breast cancer patients often suffer from CRF which frequently impairs quality of life (QoL). Despite the high prevalence of CRF in breast cancer patients and the severe impact on the physical and emotional well-being, effective treatment methods are scarce.

Physical activity for breast cancer patients has been reported to decrease fatigue, to improve emotional well-being and to increase physical strength. The pathophysiological and molecular mechanisms of CRF and the molecular-biologic changes induced by exercise, however, are poorly understood.

In the BEST trial we aim to assess the effects of resistance training on fatigue, QoL and physical fitness as well as on molecular, immunological and inflammatory changes in breast cancer patients during adjuvant radiotherapy.

**Methods/design:**

The BEST study is a prospective randomized, controlled intervention trial investigating the effects of a 12-week supervised progressive resistance training compared to a 12-week supervised muscle relaxation training in 160 patients with breast cancer undergoing adjuvant radiotherapy. To determine the effect of exercise itself beyond potential psychosocial group effects, patients in the control group perform a group-based progressive muscle relaxation training. Main inclusion criterion is histologically confirmed breast cancer stage I-III after lumpectomy or mastectomy with indication for adjuvant radiotherapy. Main exclusion criteria are acute infectious diseases, severe neurological, musculosceletal or cardiorespiratory disorders. The primary endpoint is cancer-related fatigue; secondary endpoints include immunological and inflammatory parameters analyzed in peripheral blood, saliva and urine. In addition, QoL, depression, physical performance and cognitive capacity will be assessed.

**Discussion:**

The BEST study is the first randomized controlled trial comparing progressive resistance training with muscle relaxation training in breast cancer patients during adjuvant radiotherapy. Based on the analysis of physiological, immunological and inflammatory parameters it will contribute to a better understanding of the physiological and psychosocial effects and the biological mechanisms of resistance training. The ultimate goal is the implementation of optimized intervention programs to reduce fatigue, improve quality of life and potentially the prognosis after breast cancer.

**Trial registration:**

ClinicalTrials.gov NCT01468766

## Background

Adjuvant radiotherapy is used in more than 90% of all breast cancer patients. It is usually given after breast-conserving surgery and may be given after a mastectomy if patients are at high risk of recurrence. After breast-conserving surgery, adjuvant radiotherapy to the involved breast significantly increases the progression free survival and reduces the breast cancer death rate by about a sixth [1]. While radiotherapy reduces breast cancer recurrence and mortality it may also be associated with acute and long term toxicity. The most frequently reported adverse effect is cancer-related fatigue (CRF), a common early and also a late side-effect of irradiation, reported in up to 80% of patients during radiotherapy [2-6]. As per definition, CRF is a persistent, subjective sense of tiredness related to cancer or cancer treatment that interferes with usual functioning and that is usually not relieved with rest and is not related to an excessive amount of activity. Over the course of radiotherapy the proportion of patients with CRF and the severity of CRF gradually tends to intensify. CRF peaks at the end of radiotherapy and in about 30% of patients it may persist even for many months post-treatment [3,6-8]. Despite the high prevalence and the severe impact of CRF on the physical and emotional well-being and the quality of life (QoL), the aetiology of this common symptom and its correlates are poorly understood and effective treatment methods are scarce. Several interventions have been tested in the management of CRF. Although an optimal method has not yet been established, some promising results have been reported with relaxation therapy, group psychotherapy, physical exercise and sleep. The National Comprehensive Cancer Network (NCCN) guidelines recommend treatment for pain, emotional distress, and anemia as well as optimizing treatment for sleep dysfunction, nutritional deficiency or imbalance, and comorbidities [9]. Initially tested pharmaceuticals have shown severe adverse effects (e.g. erythropoietin), or did not show efficacy in phase III studies (e.g. methylphenidate) [10]. A Cochrane review from 2008, a roundtable of the American College of Sports Medicine published in 2010, and a recent comprehensive meta-analysis on published reports of 44 exercise studies with the endpoint CRF concluded that exercise may be an effective treatment method for CRF, but that the evidence is not yet convincing [11-13]. The meta-analysis published by Brown et al, however, was based on summary data from actual research papers but did not analyze individual patient data [13]. However, most of the previously reported controlled intervention trials used “usual care” as comparison group. Therefore, it is unclear to what extent the observed effects may be based on the physical exercise itself, or rather on psychosocial factors related to the group support or the attention by the trainer. Thus, methodologically correct studies are warranted to better define the causes, the optimal prevention and the management of CRF.

Furthermore, it is still unclear what type of exercise, i.e. aerobic or resistance training, and what point in time, i.e. during or after cancer treatment, is most effective. The majority of previous controlled trials investigated aerobic exercise. Resistance training has been little examined and even fewer studies tested resistance interventions performed during adjuvant radiotherapy [11,14,15].

The molecular mechanisms of fatigue as well as the molecular changes induced by exercise are still largely unknown. Inflammation and other immunomodulatory mechanisms are supposed to be of importance for the outcome and prognosis of cancer. Irradiation can cause a weakening of the immune system but may also induce severe systemic inflammation in the short, and perhaps even long-term [16-18]. Several large trials among healthy individuals or cancer survivors reported that exercise including resistance training can lead to a reduction of markers of inflammation such as C-reactive protein (CRP) [19-24]. These results suggest that anti-inflammatory factors might mediate the beneficial effects of resistance training on fatigue during adjuvant radiotherapy.

In addition, key immunomodulators like tumor-specific CD4^+^CD25^+^ forkhead transcription factor Fox P3 (FoxP3) positive regulatory T lymphocytes also known as regulatory T-cells (Tregs) are spontaneously induced by many types of cancer [25-27]. Increased levels of FoxP3-positive Tregs in peripheral blood and tumor have been reported in patients with various types of cancer including ovarian cancer [28,29], breast cancer [30] and other tumors [27]. A lack of FoxP3-expressing T-cells can lead to autoimmune disease, whereas an abundance of FoxP3-expressing regulatory T-cells can result in immune deficiency [25]. Increased numbers of Tregs have been associated with a worse breast cancer prognosis [31-33], For example, Bates et al. reported that high numbers of FoxP3-positive Tregs were identified in patients with ductal carcinoma in situ at increased risk of relapse, and in patients with invasive breast tumors with both shorter relapse-free and overall survival [30]. In addition to their potential value in predicting disease progression and relapse, FoxP3-positive Tregs have recently been reported to be a marker for the monitoring of therapeutic response. Merlo et al suggest that FoxP3 itself is expressed in breast cancer cells, and that the expression level is associated with patient survival [34]. Whereas increased numbers of Tregs have been correlated with a worse breast cancer prognosis [30-34], exercise has been correlated with a trend towards a better prognosis [35]. This raises the question whether exercise might have an effect on the level of Tregs and whether they might be one of the molecular mediators of the beneficial effects of exercise seen in cancer patients. To date, however, immunological and molecular factors have only been minimally studied with respect to fatigue, and the effect of resistance training during radiotherapy on the Treg level in breast cancer patients is unclear.

The aetiology of fatigue during radiotherapy is also not well defined. The course and severity differ between radiotherapy- and chemotherapy-induced fatigue, which suggest different pathways [36]. Overall, methodologically optimized randomized controlled clinical trials and a better understanding of the pathophysiology and the molecular mechanisms of fatigue induced by radiotherapy as well as the mode of action of resistance training are important for evidence-based exercise recommendations for breast cancer patients during treatment.

The BEST trial is a prospective, randomized controlled intervention study in breast cancer patients during adjuvant radiotherapy exploring the effects of a 12-week supervised progressive resistance training on CRF, QoL, depression, as well as muscular strength, cardiorespiratory fitness, and body composition. Moreover, pathophysiological, molecular and immunological mechanisms of fatigue and exercise will be analyzed.

To determine the specific effects of the exercise program itself beyond potential psychosocial effects related to a supervised group-based training, patients in the control group receive a comparable training schedule, yet with group-based progressive muscle relaxation (also called Jacobson’s progressive relaxation or Jacobson’s method) [37].

## Methods/design

### Study design

**Figure 1:**
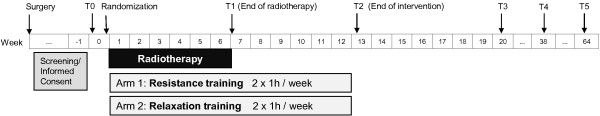
Study design of the BEST study.

**Figure 2:**
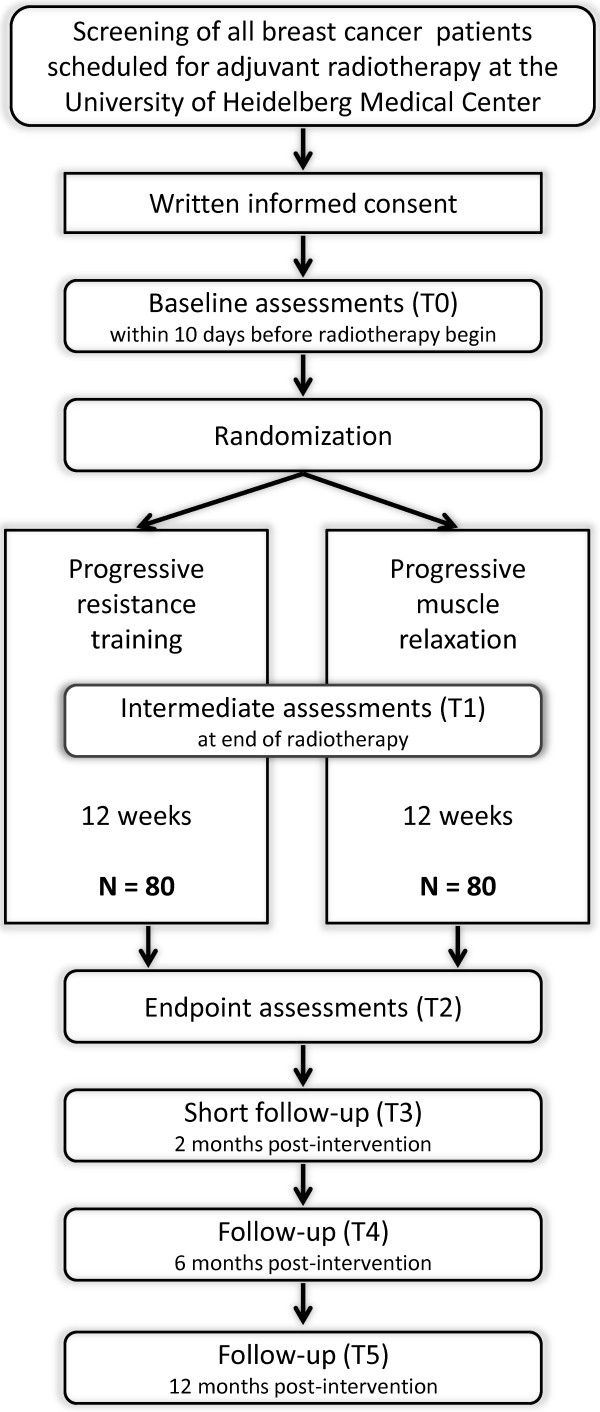
Study flow of the BEST study.

The BEST study (“Bewegung und Entspannung für Brustkrebspatientinnen unter Strahlentherapie”; English: “exercise and relaxation for breast cancer patients during radiotherapy”) is a prospective, randomized, controlled clinical intervention trial in stage I-III female breast cancer patients during adjuvant radiotherapy. Women have to provide written informed consent prior to participation in the study. After baseline assessments, participants are randomized to a supervised progressive resistance training or a supervised relaxation program over a period of 12 weeks (see Figure 1). Both interventions are administered group-based. Endpoints are assessed within 21 days before radiotherapy (baseline, T0), after the end of radiotherapy (week 7, T1), after the end of the intervention (week 13, T2), and 2, 6, and 12 months post-intervention (T3, T4, T5) (see Figure 2). Blood (serum, plasma, peripheral blood mononuclear cells (PBMCs)), urine, and saliva (5 samples over one day) are collected at T0, T1, and T2.

To enhance the participation rate and maintain high compliance to the intervention scheme, participants are offered to train for another 12 weeks in the program of their choice after completion of the 12-weeks randomized intervention period. The intervention programs and several outcome measures are based on experiences from a randomized controlled trial with breast cancer patients during chemotherapy conducted by our group (BEATE study) [38].

The BEST study has been approved by the ethics committee of the University of Heidelberg in December 2010 (number S-447/2010) and is registered at ClinicalTrials.gov (http://NCT01468766).

### Objectives

The primary objective of the BEST trial is to determine the effect of resistance training on fatigue compared to a relaxation control group among breast cancer patients during adjuvant radiotherapy.

Secondary objectives are to estimate the effects of the resistance training on quality of life, depression, cognitive function, and early and late radiotoxicity, as well as on physical fitness, including muscle strength, cardio-respiratory fitness, flexibility, and body composition. The effects of the resistance training on immunologic and inflammatory parameters and other biomarkers relevant to cancer prognosis will also be tested. Further, hypothesized biological mediators of physical activity and cancer-related fatigue will be explored and the relationships between cancer-related fatigue, physical fitness, and biomarkers of stress, inflammation, and immune function will be modelled. Safety and feasibility of progressive resistance training during radiotherapy will be evaluated, and the sustainability of the effects will be assessed.

### Patient selection

The BEST study includes women with histologically confirmed primary breast cancer who are scheduled for adjuvant radiotherapy at the University of Heidelberg Medical Center and who do not have any contraindications for a progressive resistance training. Inclusion and exclusion criteria are provided in Table 1.

**Table 1:** Inclusion and exclusion criteria of the BEST study

**Inclusion criteria**	**Exclusion criteria**
• Female patients with histologically confirmed primary breast cancer, stage I-III after lumpectomy or mastectomy scheduled for adjuvant radiotherapy at the University of Heidelberg Medical Center	• Acute infectious disease
	• Inability to walk or stand
	• Severe neurological deficiencies
• Age ≥18 years of age	• Severe cardiac or cardiovascular disease
• BMI ≥18 kg/m^2^	• Severe respiratory insufficiency
• Ability to understand and follow the study protocol	• Severe renal failure
• Willingness to come to the Heidelberg exercise facilities and adhere to study protocol	• Other concurrent malignant disease (except carcinoma in situ of skin or cervix)
• Written informed consent	• Substance abuse (potentially leading to non-compliance)
	• Participation in systematic intense resistance or aerobic training (at least 1 h twice per week)
	• Previous participation in another exercise intervention trial

### Recruitment and randomization

All eligible breast cancer patients scheduled for adjuvant radiotherapy at the University of Heidelberg Medical Center are briefly informed about the BEST study during the therapy counselling visit (about 1-2 weeks before start of radiation). If interested, patients are then informed in detail by the BEST study physician and inclusion and exclusion criteria are verified. For each patient recruited into the study, written informed consent is essential prior to inclusion into the study after extensive information about the intent of the study, the study regimen, potential associated risks and side effects. The investigator will not undertake any diagnostic measures specifically required for the clinical trial until valid consent has been obtained. Upon written informed consent, the patient is scheduled for the baseline visit, which should be within 21 days prior to the start of radiation.

After completion of the baseline assessment and if the testing procedure does not indicate cardiovascular, respiratory or neurological problems that may contraindicate resistance training, the participant is randomly allocated to one of the two intervention groups. Allocation is done by the biometrician based on a predetermined list generated with a blocked randomization SAS procedure with a fixed block size, stratified by age (< 50 / ≥ 50 years of age) and baseline physical fatigue level (< 14 / ≥ 14). Stratification is used in the randomization process, as we anticipate these variables to have major influence on the outcome. To prevent possible bias, study personnel involved in the recruitment and the baseline assessment do not have access to the randomization lists and are not aware of the block size. Conversely, the biometrician does not have influence on the recruitment procedure.

Recruitment of n=160 patients started in February 2011 and was completed in March 2013.

### Interventions

The begin of the training is the day of the first radiotherapy treatment. Patients participate in the intervention or control program for 60 minutes, twice weekly for 12 weeks. Participants train together with other cancer patients under supervision and guidance of experienced therapists. At days of radiation, participants frequently train directly before or after radiation due to logistic reasons. The physical status and well-being prior to and after a training session are recorded by the participant. The trainer documents attendance of each participant at each session. Similarly, if sessions have been missed, reasons are documented. In addition, for the resistance training individual weights and number of repetitions performed are documented.

#### Exercise intervention

Sessions are comprised of machine-based resistance exercise located at the training center of the Institute for Sports and Sports Sciences in Heidelberg. The hypothetical one-repetition maxima (1-RM) according to the Brzycki-Method [39] is defined for each exercise task in the first training session. The resistance training protocol complies with the American College of Sports Medicine (ACSM) exercise guidelines for cancer survivors [12] and with ACSM recommendations for progressive resistance training for novice weightlifters and older adults. This protocol includes one to three sets at a weight that can be handled for 8 to 12 repetitions (approximately 60–80% of 1-RM) [40,41] with a resting time of one minute between the sets. A complete session takes approximately 60 minutes and includes eight different types of exercises for major upper and lower muscle groups: 1) leg extension; 2) leg curl; 3) leg press; 4) shoulder internal and external rotation; 5) seated row; 6) latissimus pull down; 7) shoulder flexion and extension; and 8) butterfly and butterfly reverse. Training is progressive in terms of weight increase to the next machine weight level (at least by 5%) after successfully completing 3 sets of an exercise with 12 repetitions in three consecutive exercise sessions.

#### Relaxation intervention

Similar to the resistance training the relaxation intervention is performed for 60 minutes, twice weekly for 12 weeks in the exercise facility of the National Center for Tumor Diseases (NCT) in Heidelberg. It is based on the progressive muscle relaxation method according to Jacobson and does not include any aerobic or muscle strengthening components [37].

### Outcome measures

The outcome measures used in the BEST study are summarized in Table 2.

**Table 2:** Assessments and instruments used in the BEST study

**Outcomes**	**Instrument**	**T0**	**T1**	**T2**	**T3**	**T4**	**T5**
**Primary endpoint**							
Fatigue	Fatigue assessment questionnaire (FAQ)	X	X	X	X	X	X
**Secondary endpoints**							
Quality of life	EORTC QLQ30 / BR-23 questionnaire	X	X	X	X	X	X
Depression	Center for Epidemiological Studies Depression Scale (CES-D)	X	X	X	X	X	X
Cognitive function	Trail-making-test	X	X	X		X	X
Body composition	Bioimpedance analysis, weight, height, waist and hip circumference	X	X	X		X	X
Muscle strength	Isometric and isokinetic strength of representative muscle groups for upper and lower extremity measured at the IsoMed2000®	X		X		X	X
Cardio-respiratory fitness	Spiroergometry (VO2peak)	X		X		X	X
Flexibility	Range of motion measured at the IsoMed2000®	X		X		X	X
Radiotoxicity	Acute radiation dermatitis, LENT-SOMA classification for late effects, ECOG performance status, hemoglobin , and thrombocytes at end of radiotherapy		X	X			
Circulating immune cells	Analyzed in peripheral blood	X	X	X			
Biomarkers of inflammation and oxidative stress	Analyzed in peripheral blood and urine	X	X	X			
Salivary cortisol	Saliva collected at five different time points during a day	X	X	X			
Sample collection data	Date and time of collection, as well as time since last food or fluid intake, vigorous physical activity (during last 12 h), NSAID intake (during last 12 h), smoking (during last 24 h), caffeine intake (during last 6 h), alcohol intake (last 48 h), acute infections, and sleep quality during last night are recorded.	X	X	X			
Safety of training interventions	Number of participants with lymphedema, pain, nausea, dyspnea, or tachycardia during the intervention phase	at each training session					
**Others**							
Socio-demographic factors	Recording of date of birth, education, occupation, socio-familial situation, smoking, alcohol consumption	X					
Breast cancer characteristics	Family history, TNM status, grading, ER/PR status, HER2-score, p53, bcl-2, Ki-67,	X					
Medical history	Recording of pre-existing diseases and of allergies	X					
Treatment data	Pre-treatment: ECOG at diagnosis, date and type of breast surgery, affected lymph nodes, (neo-) adjuvant chemotherapy (type, last infusion), hormone therapy		X				
	Radiation: technique (3D, IMRT), type and dose, start and stop date, interruptions						
Concomitant medication	Recorded at each visit on a medication log form	X	X	X			
Physical activity history	Physical activity in adolescence, pre-diagnosis, during, and after intervention is recorded, including walking, cycling, and sports	X		X	X	X	X

#### Fatigue

The primary endpoint is change of fatigue from baseline to week 13. Fatigue is assessed with the Fatigue Assessment Questionnaire (FAQ) which is a 20-item, multidimensional self-assessment questionnaire that has been validated for a German-speaking population [42]. It covers the physical, affective, and cognitive fatigue dimensions, and includes one item on sleep disorders. Scores are derived by summing the answers (0=not at all, 1=a little, 2=quite a bit, 3=very much) of the appropriate items. Reference values of the FAQ scores are available from a representative sample of the German population including 1,340 women stratified by age [43].

#### Quality of life (QoL)

QoL is assessed with the validated 30-item self-assessment questionnaire of the European Organisation for Research and Treatment of Cancer (EORTC QLQ-C30, version 3.0). It includes five multi-item functional scales (physical, role, emotional, cognitive, and social function), three multi-item symptom scales (fatigue, pain, nausea/vomiting), and six single items assessing further symptoms (dyspnea, insomnia, appetite loss, constipation, diarrhea) and financial difficulties [44]. In addition, the 23-item breast cancer specific module (EORTC QLQ-BR23) is applied, assessing common problems of breast cancer patients, e.g. with the affected breast or arm. Scores are derived according to the EORTC scoring manual [45]. Reference values are available from the EORTC reference manual [46] and from a sample of the general German population stratified by gender and age [47]. Further, evidence-based guidelines for the interpretation of the clinical relevance of changes in the different EORTC QLQ-C30 subscales were recently published [48], categorizing differences between scores in trivial, small, medium, or large effect sizes.

#### Depression

Depressive symptoms are assessed with the 20-item Center for Epidemiological Studies Depression Scale (CES-D). The CES-D scale is a widely used validated self-report instrument to measure current depressive symptomatology and to identify possible cases of depressive disorders, both in the general population and in patients with cancer [49].

#### Cognitive function

Cognitive function (concentration, cognitive flexibility) is estimated with the trail-making-test. This is a standardized, reliable and valid measure used in neuropsychological diagnostics [50,51]. The test measures the time needed by the participant to connect numbers and letters on a sheet of paper in a logical sequence.

#### Radiotoxicity

Onset and duration of acute radiodermatitis is recorded due to the NCI-CTCAE criteria version 4.02. The “Late Effects of Normal Tissue – Subjective, Objective, Management, and Analytic scales” (LENT-SOMA) are applied at week 13 asking for ulcerations, telangiectasias, palpatory changes, retraction, atrophy, edema in the breast, lymph edema, and fibrosis [52]. The LENT-SOMA allows the quantification of late effects on normal tissue.

#### Physical fitness

All fitness measures are performed by trained study personnel at the Division of Preventive Oncology at the NCT.

*Muscle strength* is assessed by measuring isometric (4 positions) and isokinetic (2 angular velocities) muscle capacity with the IsoMed 2000® diagnostic module (isokinetic evaluation and training machine, D&R Ferstl GmbH, Hemau, Germany). The protocol includes testing of representative muscle groups for upper (shoulder rotators) and lower extremity (knee extensors and flexors). Reliability and validity of isokinetic dynamometer machines have been reported in several studies, with coefficients of variation below 10% [53-55].

*Endurance performance* (VO2peak) is measured on a bicycle ergometer (Ergostik, Geratherm Respiratory GmbH, Bad Kissingen, Germany) by performing a symptom-limited test with a step protocol (starting at 50 watt with steps of 25 watts every 2 minutes). The criteria of exhaustion is defined as achieved estimated maximum heart rate, plateau in VO2 and RQ >1.1. VO2peak is defined as highest 30-second average during the test. Peak workload, peak oxygen uptake and oxygen uptake at ventilatory threshold are taken for analysis. Cardiorespiratory exercise testing is well established in cancer patients and recommendations for testing procedures as well as safety guidelines in clinical trials with cancer populations have been defined [56]. The procedure is also used to exclude exercise-contraindicating cardiac impairments.

*Body composition* of the participants is measured with bioelectrical impedance analysis (BIA, Akern Srl, Pontassieve, Italy). This is a quick and non-invasive method, which determines the electrical impedance, or opposition to the flow of an electric current through body tissues to calculate an estimate of total body water, fat-free body mass and body fat [57]. BIA gives reliable measurements of body composition with minimal intra- and inter-observer variability in healthy volunteers [58]. In cancer patients during therapy, derived variables need to be interpreted with caution, e.g. due to potential lymphedema. In addition, algorithms used to calculate %fat mass might lead to biased values [59]. Thus, our focus will be on inter-individual changes with respect to the phase angle (reactance and resistance) during the intervention period rather than on absolute values or computed values for different compartments. In addition, body weight in light clothing, height, hip- and waist circumference are measured.

#### Biospecimen collection and biomarkers

Serum, plasma, and PBMCs are derived from whole peripheral blood samples, processed within 2 hours after taking the blood sample and stored at -80°C or cryopreserved in liquid nitrogen (PBMCs) for analyses of biomarkers after completion of the last study participant. Only CRP is directly analysed with nephelometry within the clinical routine lab.

Urine samples are collected for analyses of biomarkers of oxidative stress, i.e. urinary F2-isoprostane and 8-oxo-dG measured by chromatography-based methodology.

Saliva samples are collected with salivettes by the participants at 5 specific time points during a day (at waking, +0.5 h, noon, 5 pm, 10 pm/bed time) for analyses of diurnal cortisol slopes and cortisol morning peaks after study completion.

Immunological factors are assessed in fresh blood, including the quantity of FoxP3+ CD25+ regulatory T-cells and circulating lymphocytes subpopulations. In addition, in a subpopulation of n=40 participants (20 of each intervention arm) the specificity of FoxP3+ CD25+ regulatory T-cells is measured.

#### Safety issues

Potential adverse effects (e.g. lymphedema, pain, muscle soreness, nausea, dyspnea, tachycardia) are recorded by the participants at each training session by standardized questionnaires throughout the intervention period. Adverse events reported spontaneously by the patient or observed by physiotherapists, study nurse or physicians are recorded, e.g. sports accidents or injuries.

### Sample size

The primary aim is to compare changes on the overall fatigue scale from baseline to week 13 between the exercise and relaxation group. To detect a mean standardized effect size of 0.5 with a two-sided t-test with significance level 0.05 with a power of 80% a sample size of 80 breast cancer patients per arm is needed, 160 women in total, assuming a maximal drop-out rate of 20%. However, adjustment for the pre-intervention fatigue value in the regression models on post-intervention fatigue will lead to an improved power above 80% depending on the correlation between the pre- and post-intervention values [60].

This sample is also large enough to detect medium sized clinically relevant intervention effects on the secondary outcome EORTC QLQ-C30 subscales. Evidence-based guidelines for the interpretation of the clinical relevance of changes in the different EORTC QLQ-C30 subscales were recently published [48], categorizing difference between scores (on the 0-100 points scale) in trivial, small, medium, or large effect sizes. For example, effects are considered as medium size for differences of 19-29 in role function, differences of 14-22 in physical function, 11-15 in social function, 9-14 in cognitive function, and 13-19 for fatigue.

### Data analysis

The main intervention effect will be assessed on the basis of a comparison between exercisers and controls as defined at randomization, regardless of exercise adherence, i.e. according to the intent-to-treat principle. The differences in fatigue between groups will be assessed with a generalized estimating equation (GEE), which accounts for repeated observations on the same subjects over time. This method provides the most efficient estimate for the intervention effect in pretest-posttest trials [61]. Normality assumptions will be checked and data if necessary transformed. Imputation-based sensitivity analyses will be conducted to examine the potential effect of missing data on the results.

Similar analyses as for fatigue will also be performed for the secondary endpoints. In addition, analyses will be performed stratified by pre-treatment (e.g. neoadjuvant, adjuvant or no previous chemotherapy), to evaluate potential differential effects of the exercise intervention by pre-treatment. Further, subgroup effects of resistance training versus relaxation controls will be explored stratified by training adherence, changes in muscle strength, cardiorespiratory fitness, and body composition. Correlation analyses will be used to examine the relationship between changes of the various measured endpoints. Regression analyses regarding the repeated measurement design (T0, T1, T2, T3, T4, T5) will be applied to investigate the association between therapy modalities, cardiorespiratory fitness, muscle strength, and body composition and the different fatigue as well as QoL dimensions. The influence of other potential confounding factors, such as age, smoking, clinicopathologic characteristics, and comorbidities will be explored and accounted for in the analyses.

In addition, change in physical activity behavior post intervention will be explored for the follow-up time points using descriptive analysis.

## Discussion

The BEST study will add to current knowledge about exercise in breast cancer patients with respect to several novel aspects being tested: (1) Exercise performed in parallel to radiotherapy; (2) progressive resistance training; (3) exercise effect beyond psychosocial training effects; (4) effects on immune function, and (5) sustainability and long-term effects of a 12-week exercise intervention.

Among breast cancer patients receiving radiotherapy the most frequently reported side effect is fatigue. As about 72.000 women in Germany are newly diagnosed with breast cancer each year [62], the majority receiving radiotherapy, this radiation-related fatigue is a substantial health problem. Exercise may be an effective treatment against fatigue. Thus, it is surprising that exercise during radiotherapy has been minimally investigated in breast cancer patients so far. To our knowledge, only five randomized exercise trials included breast cancer patients during adjuvant radiotherapy [63-67]. Three of these studies included also patients during other adjuvant treatments (chemotherapy, hormone therapy) [64,66,67] and one was a pilot study including also prostate cancer patients [65], leaving only one exercise study with only breast cancer patients during adjuvant radiotherapy but with a small sample size of only n=46 [63].

Radiation can be muscle damaging (myotoxic), resulting in significant reductions in skeletal muscle mass and function [68]. Resistance training can counteract this muscle degradation. The negative influence of cancer therapy is a major rationale to investigate the effect of resistance exercise during adjuvant radiotherapy, as training in parallel to adjuvant treatment might prevent or mitigate treatment side effects such as fatigue.

Previous randomized exercise trials mostly investigated aerobic exercise, but benefits of resistance training in cancer patients and survivors on quality of life and fatigue have also been reported [15,69-71]. To our knowledge, only seven studies investigated pure resistance training in cancer patients and survivors [69,72-77]. Among those studies, two had insufficient power (n=22 and 38) [76,77] and of the others only three focused on breast cancer patients [69,72,75]. However, no randomized controlled trial investigated progressive resistance training in breast cancer patients during adjuvant radiotherapy.

A further strength of the BEST study is the choice of the control group, i.e. of a standardized relaxation training without any aerobic or resistance exercise components, but which reflects the training schedule and psychosocial conditions of the exercise intervention. Positive psychosocial “side effects” of group-based exercise training have been observed [78], which potentially can contribute to a lower perception of fatigue and higher QoL, in addition to physiological effects of the exercise on fatigue. Thus, the BEST design enables us to discern the “pure” physiological effects of exercise beyond potential psychosocial effects of a group-based training, which are related to social interactions, group support, improved self-efficacy, or attention by the trainer. Psychosocial and behavioural interventions have also shown some beneficial effects regarding fatigue and QoL [79,80]. Thus, it is still unclear, to what extent the observed benefits of exercise interventions are really caused by physical training, because previous studies have commonly used a “usual care” control group.

Further, the pathophysiology of fatigue and the mode of action of exercise on its prevention and treatment are not well understood. Our trial enables investigation of the effects of resistance training on immunologic parameters as well as on biomarkers of inflammation, oxidative stress, and diurnal cortisol slopes. While the intervention effect on fatigue and potential underlying biological mediators is one focus of the trial, another focus is the examination of the effects of resistance exercise on prognostic factors and health-relevant biomarkers. Especially regulatory T-cells will be investigated in detail, as those have been found to be associated with prognosis in breast cancer patients [31-33].

Finally, in case of the detection of beneficial effects during or at the end of an exercise intervention, it is of interest whether those benefits sustain over a longer period of time. To-date, data on the sustainability of exercise interventions is limited. Therefore, we follow the BEST participants over one year post-intervention and assess at 3 post-intervention time-points fatigue, QoL, physical fitness, and their physical activity behavior.

In summary, the BEST study shall contribute to a better understanding of the physiological and psychological effects of resistance training and their biological and immunological mechanisms in breast cancer patients during adjuvant radiotherapy. The ultimate goal is the implementation of an optimized intervention program to reduce fatigue and improve quality of life and potentially the prognosis after breast cancer.

## Abbreviations

ACSM: American college of sports medicine; BIA: Bioelectrical impedance analysis; BMI: Body mass index; CES-D: Center for epidemiological studies depression scale; CRF: Cancer related fatigue; CRP: C-reactive protein; FAQ: Fatigue assessment questionnaire; FoxP3: CD4^+^CD25^+^ forkhead transcription factor Fox P3 regulatory T lymphocytes (Treg cells); GEE: Generalized estimating equation; IMRT: Intensity-modulated radiation therapy; NCCN: National comprehensive cancer network; NCT: National center for tumor diseases; NSAID: Non-steroidal anti-inflammatory drug; PBMCs: Peripheral blood mononuclear cells; QoL: Quality of life; RM: Repetition maxima; Tregs: Tumor-specific regulatory T-lymphocytes

## Competing interests

The authors declare that they have no competing interests.

## Authors’ contributions

KS, JW, KP, MES and CMU conception, design, trial protocol and initiation of the project; PB conception and supervision of immunological analyses; NH, CMU and MES supervision of biospecimen collection and analyses; JW conception and supervision of training interventions and physical performance diagnostics; OK study coordinator, performs endpoint assessments; KP and HH study physicians; MES and KS study and data management; KP, MES and KS drafted and finalized the manuscript. JD medical advice. All authors have read and approved the final manuscript.

## Pre-publication history

The pre-publication history for this paper can be accessed here:

http://www.biomedcentral.com/1471-2407/13/162/prepub
